# A Prognostic Model for Colon Adenocarcinoma Patients Based on Ten Amino Acid Metabolism Related Genes

**DOI:** 10.3389/fpubh.2022.916364

**Published:** 2022-05-27

**Authors:** Yangzi Ren, Shangwen He, Siyang Feng, Wei Yang

**Affiliations:** ^1^Department of Pathology, Guangdong Province Key Laboratory of Molecular Tumor Pathology, School of Basic Medical Sciences, Southern Medical University, Guangzhou, China; ^2^The First School of Clinical Medicine, Southern Medical University, Guangzhou, China; ^3^Department of Thoracic Surgery, Nanfang Hospital, Southern Medical University, Guangzhou, China; ^4^Research Department of Medical Sciences, Guangdong Provincial People's Hospital, Guangdong Academy of Medical Sciences, Guangzhou, China

**Keywords:** colon adenocarcinoma, amino acid metabolism, prognostic model, immune checkpoint, immune therapy

## Abstract

**Background:**

Amino acid metabolism plays a vital role in cancer biology. However, the application of amino acid metabolism in the prognosis of colon adenocarcinoma (COAD) has not yet been explored. Here, we construct an amino acid metabolism-related risk model to predict the survival outcome of COAD and improve clinical decision making.

**Methods:**

The RNA-sequencing-based transcriptome for 524 patients with COAD from The Cancer Genome Atlas (TCGA) was selected as a training set. The integrated Gene Expression Omnibus (GEO) dataset with 1,430 colon cancer samples was used for validation. Differential expression of amino acid metabolism-related genes (AAMRGs) was identified for prognostic gene selection. Univariate cox regression analysis, LASSO-penalized Cox regression analysis, and multivariate Cox regression analysis were applied to construct a prognostic risk model. Moreover, the correlation between risk score and microsatellite instability, immunotherapy response, and drug sensitivity were analyzed.

**Results:**

A prognostic signature was constructed based on 10 AAMRGs, including ASPG, DUOX1, GAMT, GSR, MAT1A, MTAP, PSMD12, RIMKLB, RPL3L, and RPS17. Patients with COAD were divided into high-risk and low-risk group based on the medianrisk score. Univariate and multivariate Cox regression analysis revealed that AAMRG-related signature was an independent risk factor for COAD. Moreover, COAD patients in the low-risk group were more sensitive to immunotherapy targeting PD-1 and CTLA-4.

**Conclusion:**

Our study constructed a prognostic signature based on 10 AAMRGs, which could be used to build a novel prognosis model and identify potential drug candidates for the treatment of COAD.

## Introduction

Colon adenocarcinoma (COAD) is the most common type of colorectal cancer (1). According to the Global Cancer Observatory (GCO) (gco.iarc.fr) in 2020, there were an estimated 1.4 million new cases of colon cancer and 0.5 million deaths worldwide (2). Late diagnosis and lack of reliable biomarkers account for the poor prognosis of COAD (3). Despite many efforts, the attempt to use a single biomarker to predict the outcome of COAD has been unsuccessful (4).

Metabolic reprogramming is a common feature of tumor cells, which is crucial for rapid tumor growth and adaption to tumor microenvironment (5, 6). Apart from the well-known Warburg effect, metabolic alterations in lipid and amino acid metabolism have been observed in a number of tumors, including colorectal cancer, lung cancer, and breast cancer (7). Mounting evidence have indicated that changes in amino acid metabolism contributed to the metastasis, proliferation, angiogenesis, and drug resistance of colorectal cancer (8–11). Recent study has demonstrated that inhibition of ASCT2 (function as a glutamine transporter) exerted a great anti-tumor effect in colorectal cancer (12). Meanwhile, new insights into the metabolic signatures of tumors have revealed the potential of risk prediction model, which is based on the amino acid metabolism-related genes (AAMRG) (13, 14). In addition, amino acid metabolism plays an important role in regulating tumor immunity and targeting amino acid metabolism may help to overcome immunotherapy resistance and improve existing therapies for COAD patients. Therefore, targeting the amino acid metabolism might provide novel ideas for the diagnosis and management of COAD.

## Materials and Methods

### Data Acquisition

The COAD cohort's transcriptional dataset with matching clinical information were downloaded from The Cancer Genome Atlas (TCGA) (https://cancergenome.nih.gov/). Total 524 mRNA expression profiles including 482 COAD tissues and 42 normal colon tissues were enrolled. Three datasets with 1,430 patients with colon cancer from Gene Expression Omnibus (GEO), including GSE39582 (15), GSE29621 (16), and GSE17536 (17) were selected to verify the results of TCGA data analysis (https://www.ncbi.nlm.nih.gov/geo/). The “sva” software package in R version (4.0.2) was devoted to remove the batch effects.

### Differentially Expressed AAMRGs in TCGA

Total 374 AAMRGs were extracted from the amino acid metabolism-related genes dataset (REACTOME_METABOLISM_OF_AMINO_ACIDS_AND_DERIVATIVES), which were recorded in Molecular Signatures Database (13, 18). Then, total 327 common expressed AAMRGs in TCGA and GEO were selected. Subsequently, differentially expressed AAMRGs between the COAD and control groups were analyzed using the “limma” software package in R version (4.0.2) (based on |log_2_FC| > 1 and false discovery rate <0.05).

### Construction and Validation of Prognostic Risk Score Model

The univariate Cox regression analysis was conducted for prognosis-related AAMRGs screening. The LASSO algorithm was executed to avoid overfitting the model. The multivariate Cox regression was conducted to get the optimal prognostic genes for the model. Finally, the stable AAMRGs, as the final prognosis model was constructed. We used the following equation to calculate the risk score, which was combined by regression coefficients and expression values of each AAMRG. Risk score = (index gene1 × expression of gene1) + (index gene2 × expression of gene2) + … + (index gene10 × expression of 10). All COAD patients in TCGA were divided into two subgroups (high- risk group and low-risk group) according to the median risk score. Kaplan-Meier curves were used to determine the differences in prognosis between the different groups. Finally, the first, third, fifth-year survival proportions of patients were calculated using the ROC curve. Then, the prognosis model was validated in the above merge GEO dataset.

### Establishment of Nomogram Prognosis Prediction Model

We combined age, TNM stage and risk scores to plot a nomogram model using the “rms” software package in R version (4.0.2). The calibration curves were built to show the agreement between the nomogram-predicted survival probabilities and the actual survival probabilities at first-, third-, and fifth-year.

### Gene Set Enrichment Analysis Between High-Risk Group and Low-Risk Group

To reveal the effect of differential expression of AAMRGs on biological pathways in COAD, Gene Set Enrichment Analysis (GSEA) was introduced to extract the potential biological function (19). Firstly, “c2.cp.kegg.v7.1.symbols.gmt” set was downloaded from Molecular Signatures Database. Secondly, “GSEA” software was applied to identify the enriched pathways in the two subgroups. The “ggplot2” software package in R version (4.0.2) was employed to visualize the top five significantly enriched biological processes in each subgroup.

### Association Between Different Subgroups and Somatic Variation

Mutation data of COAD was downloaded from TCGA. The tumor mutation burden (TMB) value in each COAD patient was calculated the number of mutations in each patient. Then, differences in TMB values were analyzed between high-risk group and the low-risk group. The association between the risk score and TMB level was detected using Spearman correlation coefficient. Finally, the “maftools” software package in R version (4.0.2) was applied to visualize the top 20 most frequently mutated genes in each group.

### Correlations Between Immune Cell Infiltration Between High-Risk Group and Low-Risk Group

The CIBERSORT was performed to analyse the tumor immune microenvironment of COAD. CIBERSORT (https://cibersort.stanford.edu/) is a method of enumeration of 22 immune related cell subsets, which including naïve and memory B cells, seven types of T cell, myeloid cells, NK cells, and plasma cells (20). The “CIBERSORT” software package in R version (4.0.2) was applied to analyse the proportion of 22 immune cells between high-risk group and low-risk group. Bar plot was applied to visualize the differences in immune cells between high-risk group and low-risk group.

### Differences in Immunotherapy Sensitivity Between High-Risk Group and Low-Risk Group

The immunotherapy sensitivity data of patients with COAD was obtained from The Cancer Immunome Atlas (TCIA, https://tcia.at/) which included the effectiveness score of patients with PD-1 and CTLA-4 inhibitors. Then, we detected the differences of immunotherapy sensitivity score between two subgroups.

### Sensitivity Prediction of Anticancer Drugs

The prediction of the difference in drug sensitivity between high-risk group and low-risk subgroups in COAD was conducted using the “pRRophetic” software package in R version (4.0.2) based on Cancer Genome Project (CGP),which including 138 anticancer drugs (21).

### Statistical Analysis

GraphPad 8.0 software and R version (4.0.2) were applied to analyse and visualize the statistical profile. The univariate Cox regression analysis, LASSO algorithm, and the multivariate Cox regression analysis were used to narrow down the number of candidate genes (22, 23). The “survival R” and “surviviner R” software package in R version (4.0.2) were used for Kaplan-Meier analysis. We compared the two groups by student's test or Wilcoxon test. The Pearson or Spearman correlation test was utilized to evaluate the correlations between variables. The *P* < 0.05 was considered statistically significant.

## Results and Discussion

### Identifying Differential Expressions of AAMRGs Between COAD and Normal Tissues From the TCGA Dataset

**Figure 1:**
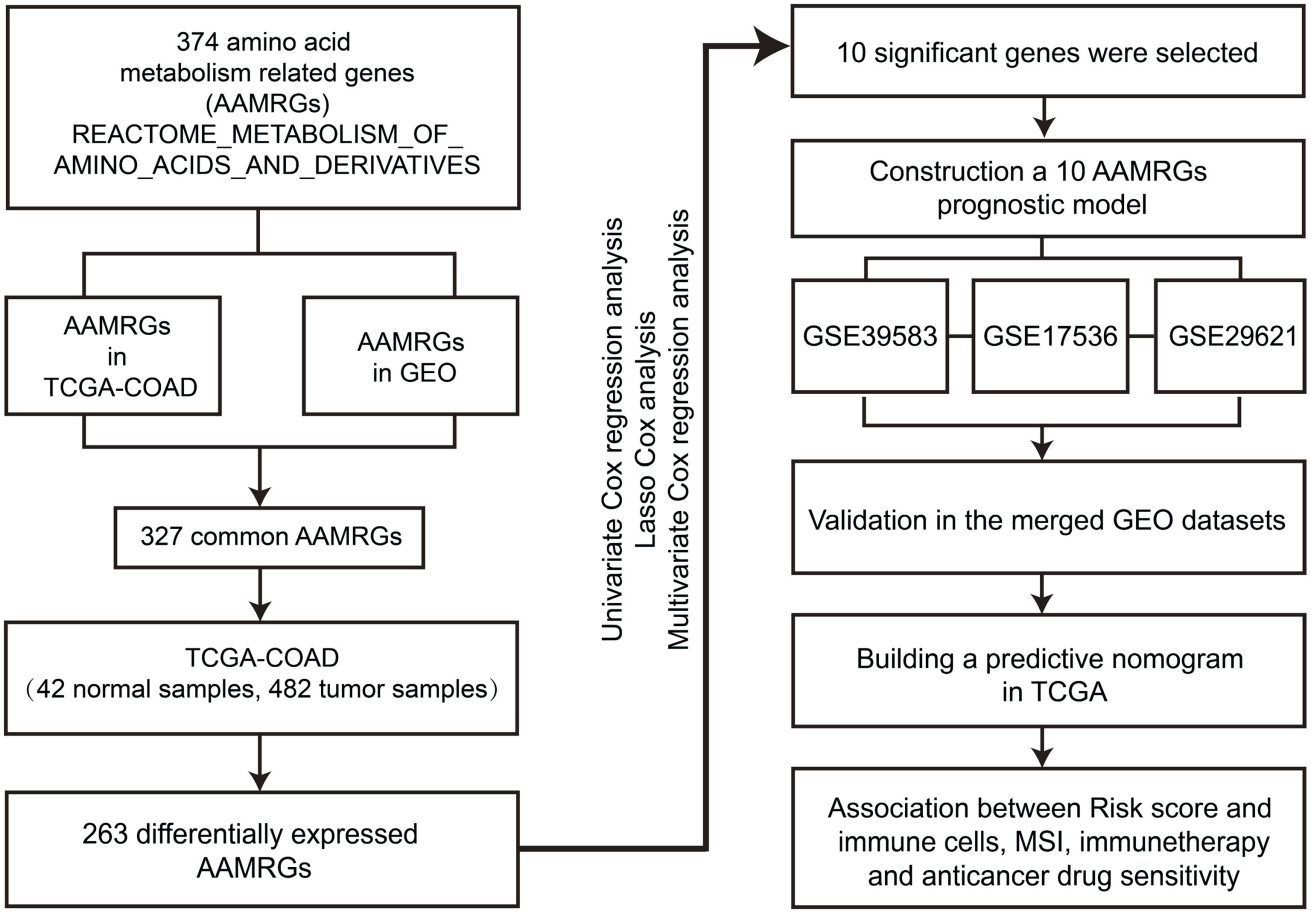
The workflow of the study.

**Figure 2:**
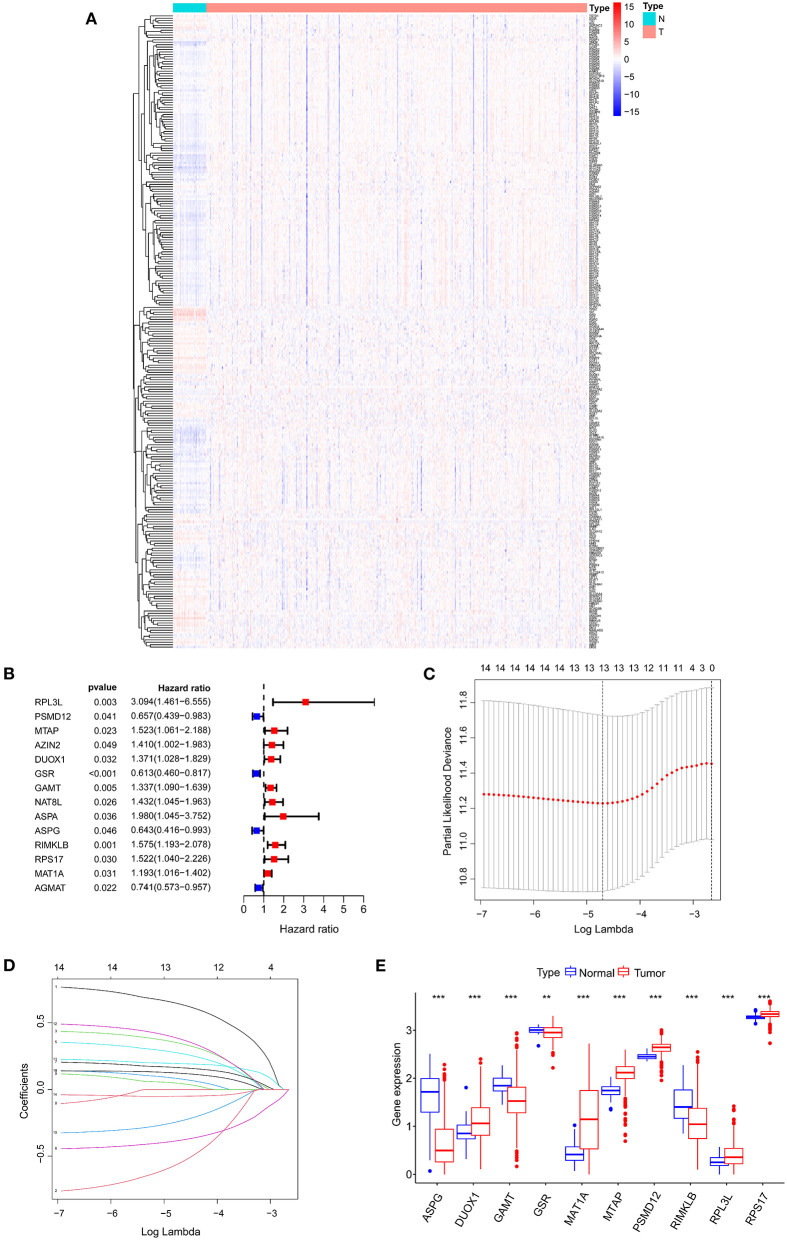
Identifying candidate genes associated with the prognosis of COAD patients. **(A)** Differential expression heatmap of amino acid metabolism-related genes in COAD and normal tissues from the TCGA. **(B)** Univariate Cox regression analysis of AAMRGs. **(C)** Turning optimal parameter (*lambda*) screening in the LASSO model. **(D)** LASSO coefficient profiles of the prognostic genes. **(E)** Box plot of mRNA expression of AAMRGs. **P < 0.01 and ***P < 0.001.

The workflow of the study is displayed in Figure 1. Total 263 differentially expressed AAMRGs (180 upregulated genes and 83 downregulated genes) in TCGA dataset were identified. The heatmap exhibited the differentially expressed AAMRGs (Figure 2A).

### Construction of Prognosis-Risk Signature Based on 10 AAMRGs

Fourteen AAMRGs were identified as prognostic related genes based on the univariate Cox regression analyses (Figure 2B). Finally, 10 prognostic related AAMRGs was screened for AAMRGs-risk signature after the LASSO analysis and the multivariate Cox regression analysis (Figures 2C,D). The boxplot showed the expression of 10 prognostic related AAMRGs in TCGA including ASPG (asparaginase), DUOX1 (dual oxidase 1), GAMT (guanidinoacetate N-methyltransferase), GSR (glutathione-disulfide reductase), MAT1A (methionine adenosyltransferase 1A), MTAP (methylthioadenosine phosphorylase), PSMD12 (proteasome 26S subunit, non-ATPase 12), RIMKLB (ribosomal modification protein rimK like family member B), RPL3L (ribosomal protein S17), and RPS17 (ribosomal protein L3 like) between COAD and normal samples (Figure 2E).

Three AAMRGs including PSMD12, MAT1A and DUOX1 were identified asprotective factors in the prognostic model. Meanwhile, seven AAMRGs including ASPG, GAMT, GSR, MTAP, RIMKLB, RPL3L and RPS17 were identified as risk factors in the prognostic model. The risk score of each COAD patient in TCGA was assessed through the equation: Risk score = (−0.33 × ASPG expression) + (0.37 × DUOX1 expression) + (0.22 × GAMT expression) + (−0.47 × GSR expression) + (0.15 × MAT1A expression) + (0.48 × MTAP expression) + (−0.81 × PSMD12 expression) + (0.25 × RIMKLB expression) + (0.65 × RPL3L expression) + (0.45 × RPS17 expression). Finally, the COAD patients in TCGA were divided into two groups (high-risk and low-risk group) based on the median risk score.

**Figure 3:**
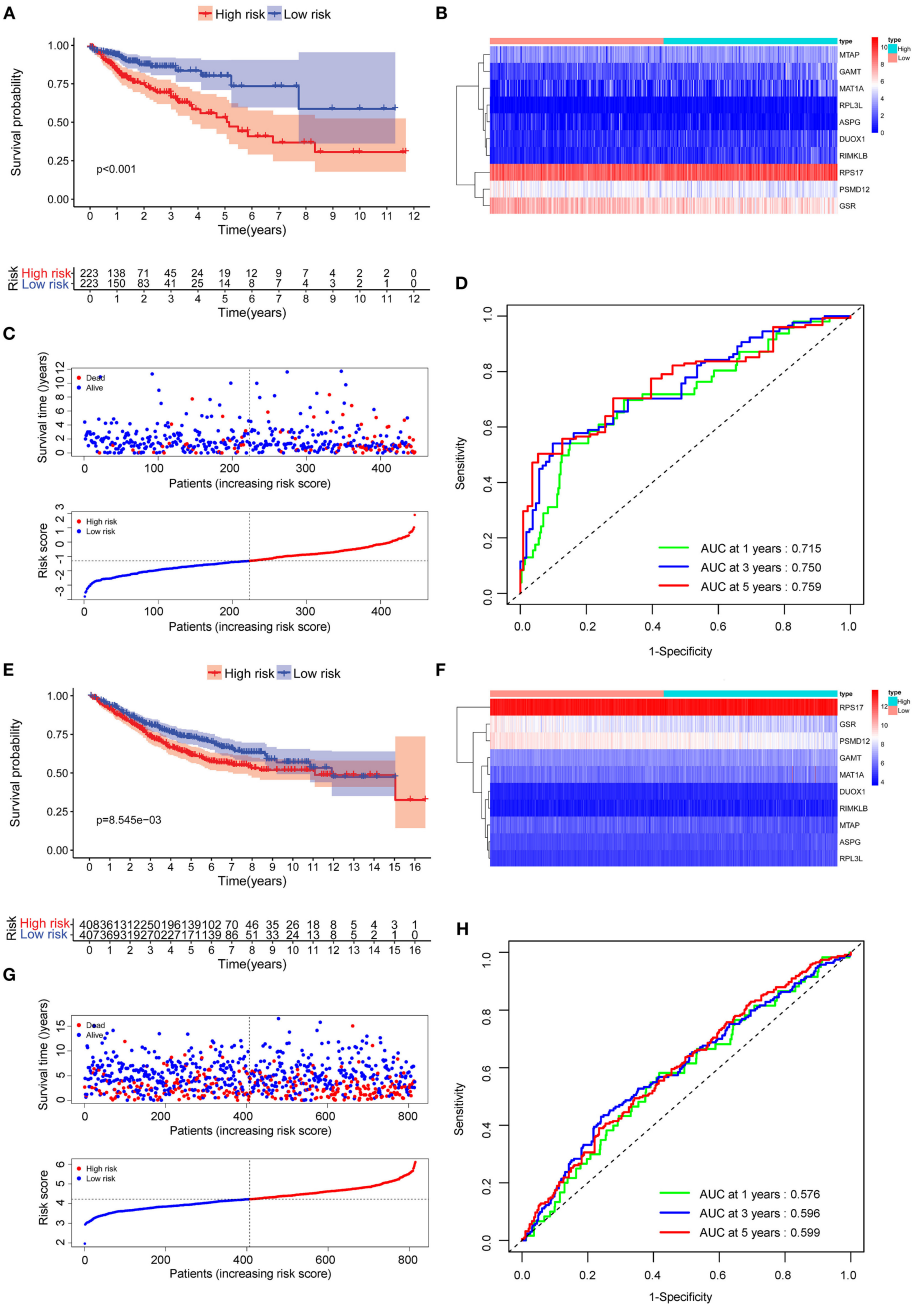
Evaluation of the prognostic performance of the AAMRGs signature in the TCGA dataset and GEO dataset. **(A)** The Kaplan-Meier survival curves of the AAMRGs signature in TCGA dataset. Patients from the TCGA dataset were stratified into two groups according to the median risk scores. **(B,C)** The distribution of risk score, survival outcomes, and the expression pattern of 10 AAMRGs in the TCGA dataset. **(D)** The ROC curves for predicting the 1-, 3-, and 5-year OS in the TCGA dataset. **(E)** The Kaplan-Meier survival curves of the AAMRGs signature in GEO dataset. Patients from the GEO dataset were stratified into two groups according to the median risk score. **(F,G)** The distribution of risk score, survival outcomes, and the expression pattern of nine AAMRGs in the GEO dataset. **(H)** The ROC curves for predicting the 1-, 3-, and 5-year OS in the GEO dataset.

The Figures 3A,C conferred a better prognosis and longer survival time in patients with COAD in the low-risk group and worse prognosis with shorter survival time in the low-risk group (Figure 3B). In addition, the result of ROC curve analysis showed that the area under the curve (AUC) of first, third, fifth-year survival was 0.715, 0.750, and 0.759, which indicated a good sensitivity and specificity in predicting the prognosis of COAD based on 10 AAMRGs (Figure 3D). To further validate the accuracy and sensitivity of the prognosis risk signature, the above merged GEO dataset was used as an external validation dataset. Consistently, a difference in OS between high-risk and low risk group was observed (*P* < 0.05) (Figures 3E–G). The AUC of first, third, fifth-year survival was 0.576, 0.596, and 0.599 (Figure 3H).

### Relationship Between the Clinicopathological Characteristics and Risk Score

**Figure 4:**
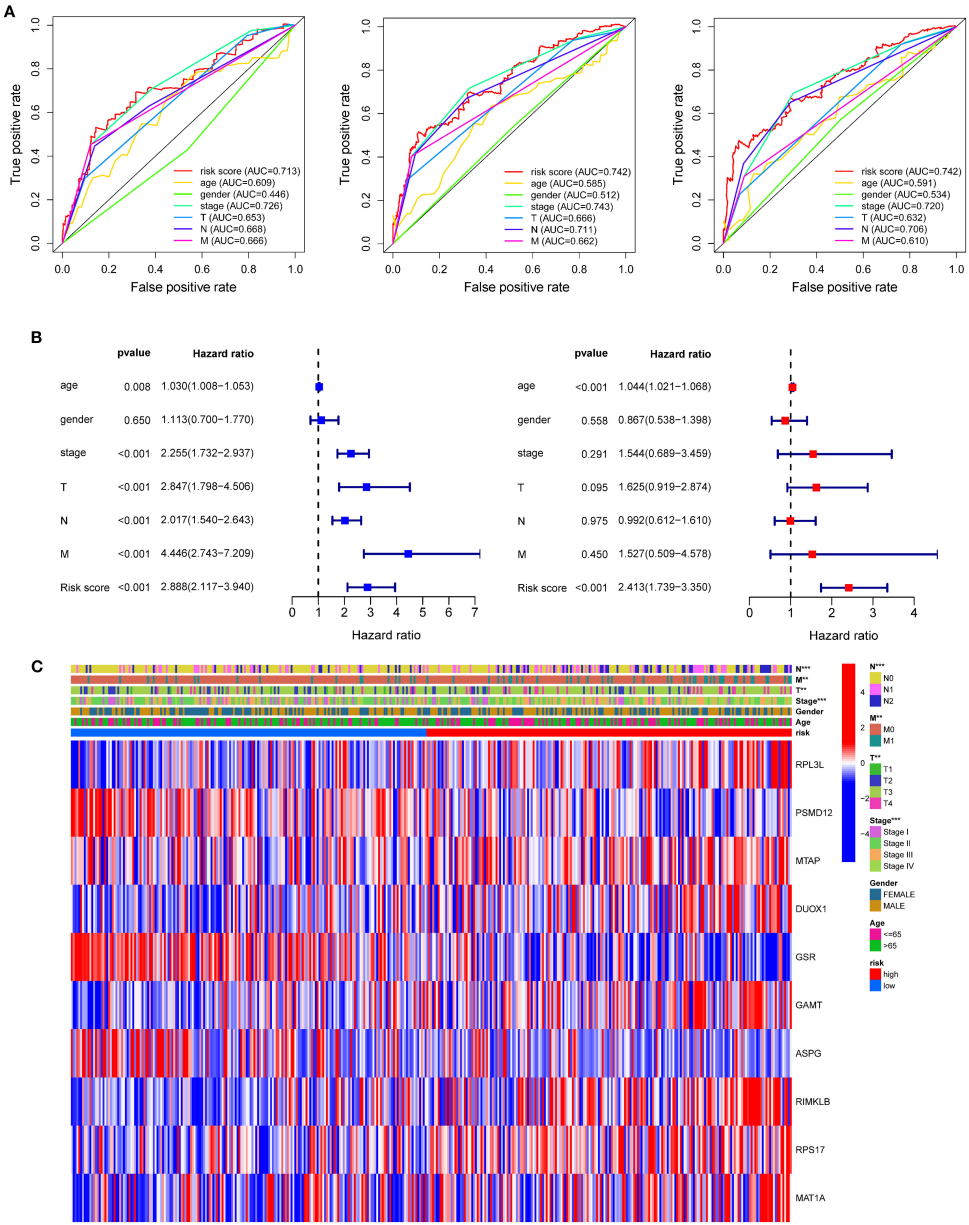
The predictive efficacy of the risk model and the relationship between risk score and clinical outcome, pathological characteristics, and prognostic value of COAD. **(A)** ROC curve to evaluate the predictive efficacy of the risk model. **(B)** Univariate Cox regression analysis and multivariate Cox regression analysis of clinicopathological characteristics and risk score. **(C)** The relationship of clinicopathological characteristics and risk scores between high- and low-risk groups from TCGA dataset. **P < 0.01 and ***P < 0.001.

Compared with the clinicopathological characteristics, the AAMRG-related prognostic risk model showed better capability in predicting one-, three-, and five-year overall survival (OS) (Figure 4A). Subsequently, the univariate Cox and multivariate Cox regression analyses revealed that the AAMRG-related prognostic risk model was an independent predictor of COAD prognosis (Figure 4B). The expression levels of the 10 screened AAMRGs and clinicopathological characteristics between high-risk and low-risk group are depicted by heatmaps. Interestingly, there was a difference in the risk tumor stages, T, N, and M stage between high-risk and low-risk groups (Figure 4C). Notably, the COAD female patient in T3–T4 stage, N1–N2 stage, M1 stage, and Stage III–IV in high-risk group showed worse survival (Supplementary Figures S1A–F). These results indicated that the risk model may have high sensitivity and specificity for COAD patients.

### Development and Evaluation of a Risk-Nomogram Based on the AAMRGs for Predicting OS in COAD Patients

**Figure 5:**
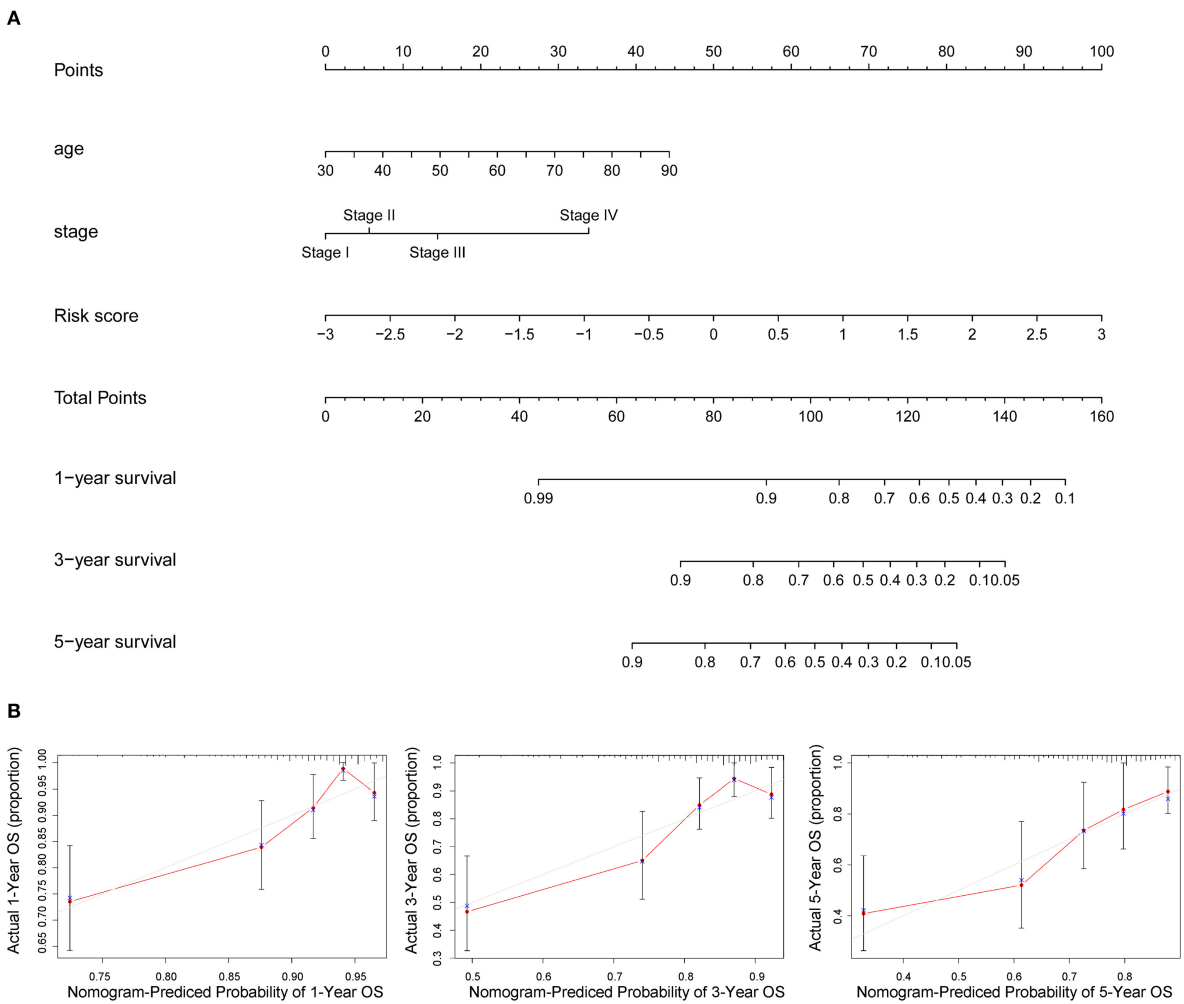
Prognostic nomogram incorporating the risk score model and clinicopathological characteristics. **(A)** The nomogram of age, stage, and risk score for predicting 1-, 3-, and 5-year survival. **(B)** The 1-, 3-, and 5-year calibration curves of TCGA dataset.

The nomogram of age, stage, and risk score based on 10 AAMRGs was constructed to predict first-, third-, and fifth-year survival (Figure 5A). The calibration curve in Figure 5B demonstrated that the prediction and actuality of fifth-year survival values were in good agreement.

### GSEA With the Prognostic Risk Signature

The GSEA was conducted between the high-risk and low-risk group based on the prognosis-risk scores. As displayed in Supplementary Figure S2A, the melanoma related signal pathway, ECM receptor interaction, WNT signaling pathway, mTOR signaling pathway and *TGF-*β signaling pathway might be positively correlated with the higher risk scores in COAD patients. In addition, the porphyrin and chlorophyll metabolism, proteasome, pentose and glucuronate interconversions, citrate cycle, TCA cycle, and drug metabolism related signal pathway were negatively correlated with COAD patients in high-risk group.

### The Correlations Between Tumor Microenvironment Cell Infiltration Characteristics and Risk Score

**Figure 6:**
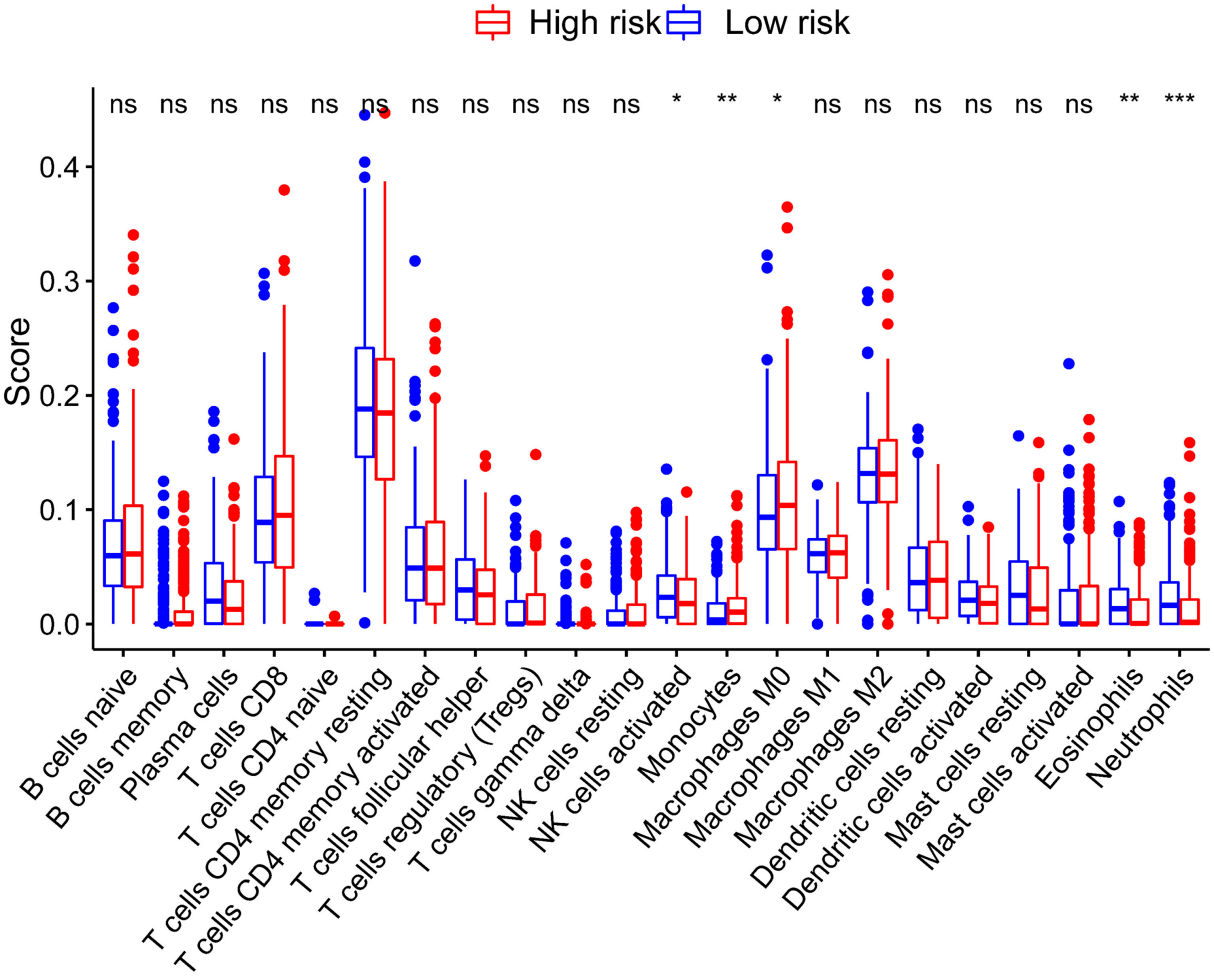
Correlations between the risk score model and tumor-infiltrating immune cells. *P < 0.05; **P < 0.01; ***P < 0.001.

The CIBERSORT results showed that activated NK cells, eosinophils, and neutrophils were more abundant in patients in the low-risk group (*P* < 0.05) (Figure 6). On the other hand, the infiltration of monocytes increased significantly in the high-risk group.

### Correlations Between the Risk Score Model and Somatic Variants

The TMB levels were calculated between high-risk and low-risk groups. However, there was no significant difference in TMB between the two groups (Supplementary Figure S2B). However, the somatic mutations of TTN, SYNE1, PIK3CA, MUC16, FAT4, ZFHX4, RYR2, OBSCN, DNAH5, PLCO were more common in the low-risk group, whereas the mutation frequency of APC, TP53, and KRAS mutations was higher in the high-risk group (Supplementary Figures S2C,D).

### Risk Score Predicts Resistance to Immunotherapy

**Figure 7:**
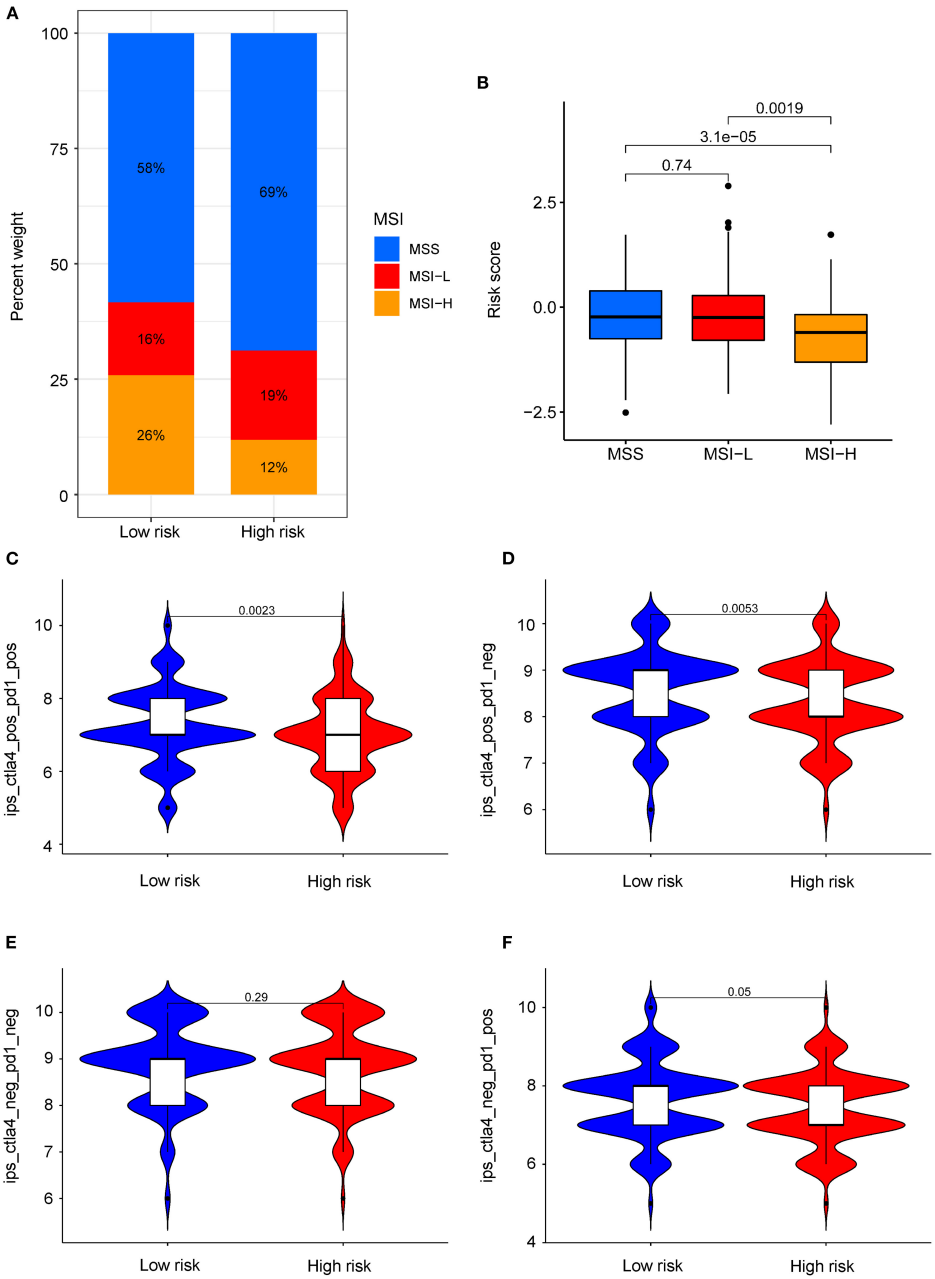
Role of risk score in predicting MSI and immunotherapy benefits. **(A)** The proportion of different MSI levels in the subgroups with high and low risk score. **(B)** Differences in risk score among groups with different MSI levels. **(C–F)** Sensitivity of patients with high and low risk score subgroups to four treatments. **(C)** PD-1 inhibitor in combination with CTLA-4 inhibitor. **(D)** CTLA-4 inhibitor alone. **(E)** Without immune checkpoint inhibitors. **(F)** PD-1 inhibitor alone.

The COAD Patients from TCIA database were divided into three groups based on the MSI status: high microsatellite instability (MSI-H), microsatellite-stable (MSS), and low microsatellite instability (MSI-L). As shown in Figure 7A, the proportion of MSI-H patients in low-risk group (26%) was higher than the high-risk group (12%). The results showed that patients in the MSI-L and MSS groups had higher risk scores, compared with MSI-H group (*P* < 0.05) (Figure 7B). This indicated that patients with lower risk scores were more sensitive to immunotherapy. Notably, the COAD patients in low-risk group were more sensitive to the combination of PD-1 and CTLA-4 inhibitors (*P* < 0.05) or CTLA-4 inhibitors alone (*P* < 0.05) than in the high-risk group (Figures 7C,D). However, there was no difference observed in the sensitivity PD-1 inhibitors alone between the two subgroups (Figures 7E,F). These data suggest that the risk score of COAD patients may affect the immunotherapy selection in COAD patients.

### Results of Risk Score Model and Drug Sensitivity

**Figure 8:**
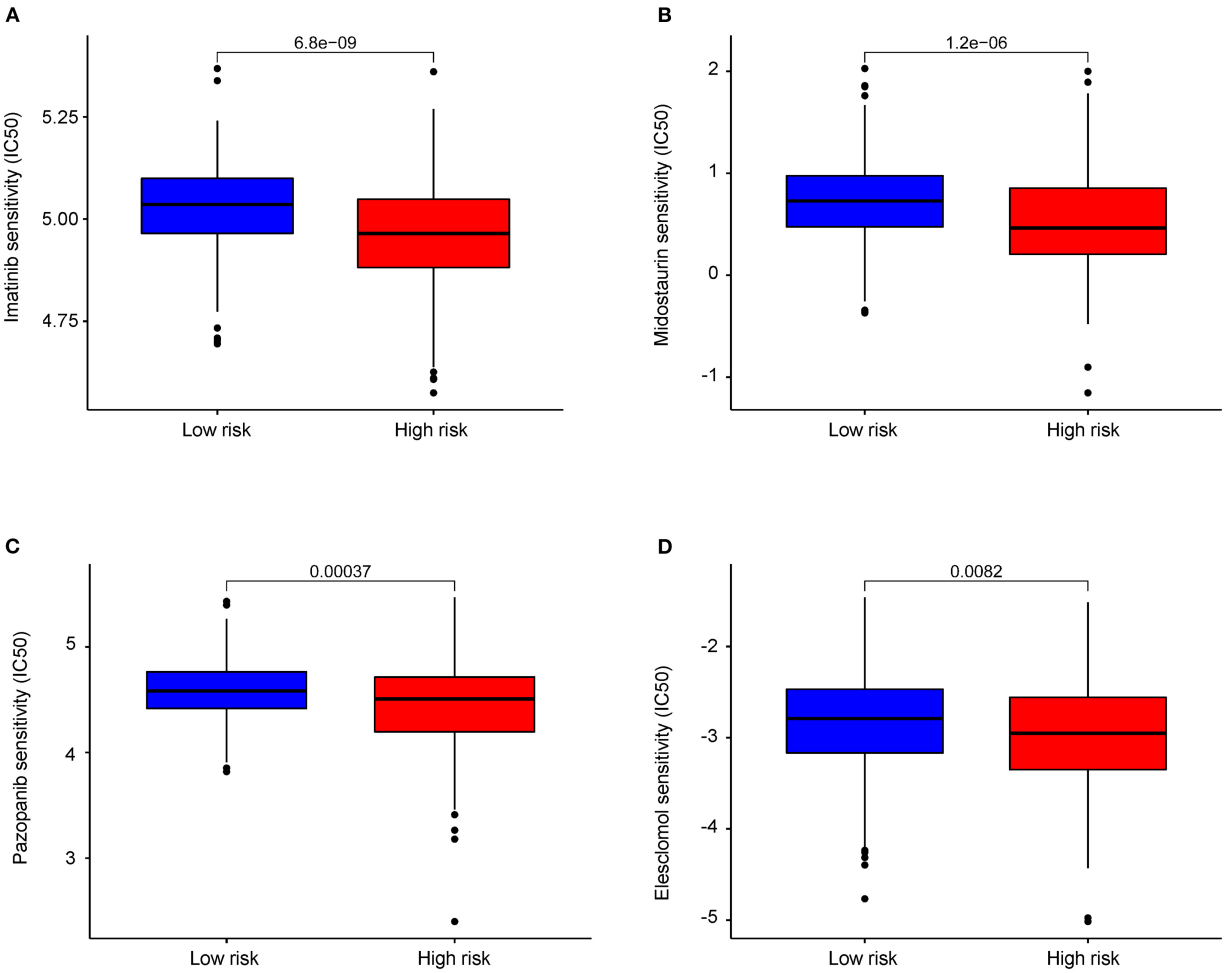
Differential chemotherapeutic response based on IC50 between the high- and low-AAMRGs-risk groups. **(A–D)** The half-maximal inhibitory concentration (IC50) of 4 chemotherapeutic agents (imatinib, midostaurin, pazopanib and elesclomol).

Four drugs including imatinib (*P* = 6.8e−09, Figure 8A), midostaurin (*P* = 1.2e−06, Figure 8B), pazopanib (*P* = 3.7e−04, Figure 8C), and elesclomol (*P* = 8.2e−03, Figure 8D) were identified with lower IC50 level in high-risk group of COAD patients, which are suggestive of better efficacy. Besides, we found that COAD patients in the low-risk group were more sensitive to drugs including paclitaxel, metformin, rapamycin, bortezomib, sorafenib, and gemcitabine (Supplementary Figures S3A–F).

## Discussion

Colorectal cancer is one of the most common types of malignant tumors. The majority of COAD patients in high-risk stage II and stage III were recommended to receive surgery combined with adjuvant chemotherapy to reduce the risk of recurrence. However, approximately half of the patients in the early stage received radical surgery developed recurrence and metastasis (24). Therefore, there is still an urgent need for constructing a prognostic model that provides personalized prognosis and precision medicine for COAD patients.

Characteristic metabolic changes in malignant cells were observed including abnormalities in amino acid metabolism, increased fatty acid synthesis and glucose uptake (25). The reprogramming of amino acids played an essential role in the tumorigenesis and tumor progression. The abnormalities in amino acid metabolism also deeply reshaped the tumor microenvironment, especially the function of immune cells (26). Several studies have demonstrated that amino acid metabolism could be a therapeutic target in multiple solid tumors (27–33). Therefore, we constructed and validated a predictive model based on AAMRGs to predict the prognosis of COAD patients.

In this study, we analyzed the differentially expressed genes related to amino acid metabolism in TCGA by performing the univariate Cox regression analysis, LASSO algorithm, and multivariate Cox regression analysis. Ten AAMRGs (ASPG, DUOX1, GAMT, GSR, MAT1A, MTAP, PSMD12, RIMKLB, RPL3L, and RPS17) were screened to construct a prognosis risk model for prediction. GSR was downregulated in COAD and inhibited the metastasis of colon cancer cell (34). In addition, GSR plays an important role in the conversion of GSSG to GSH in COAD (35). MTAP was upregulated in COAD and could accelerate the proliferation, invasion and migration of COAD (36, 37). However, the functional role of PSMD12, MAT1A, ASPG, GAMT, RIMKLB, RPL3L, and RPS17 in COAD remains unknown. The accuracy and sensitivity of the model were further validated with a merged GEO dataset. Our results indicated that high-risk is linked to worse prognosis.

To explore the correlations between immune cell infiltration and risk score, we use CIBERSORT algorithm and estimate the difference in immune infiltration between two subgroups. We found that the levels of monocytes, activated NK cells, eosinophils, and neutrophils were significantly increased in the low-risk group. These results suggested that the amino acid metabolism-related gene signature may affect the infiltration of immune cells and potentially the response of immunotherapy.

It was known that TMB is associated with the production of neoantigens and the response of immunotherapy in various tumors (38). There was only a small population of COAD patients who benefited from immunotherapy (39–42). Currently, TMB and MSI are the best predictors of the therapeutic effects of immune checkpoint inhibitors (ICIs) in COAD patients (43, 44). We further analyzed the relationship between the risk score and MSI. The low-risk group was found to have higher MSI level and increased sensitivity to immunotherapy. Data in the TCIA database showed that patients with low risk score might show greater sensitivity to the combination of PD-1 and CTLA-4 inhibitors and CTLA-4 inhibitors alone. In conclusion, dual CTLA-4/PD-1 blockade might be considered as suitable drug for patients with low-risk scores.

The combination of traditional chemotherapy drugs and targeted therapy has been widely used in the treatment of advanced colon cancer. We found that patients in the high-risk group had a higher sensitivity to elesclomol, midostaurin, pazopanib, and imatinib than in the low-risk group. Elesclomol, a reactive oxygen species (ROS) inducer, plays an important role in mediating cuproptosis (45). Pazopanib is a multi-targeted receptor tyrosine kinase inhibitor that selectively restraining the autophosphorylation of receptors such as VEGFR-2, Kit, and PDGFR-β in renal cell carcinoma (46). In our study, we may provide a new complementary for the treatment of advanced colon cancer. However, the results of this drug screening still need further validation in clinical trials.

## Conclusions

In summary, we constructed a prognostic signature based on 10 AAMRGs with strong predictive value. This study paved the way for personalized treatment of COAD patients.

## Data Availability Statement

The datasets presented in this study can be found in online repositories. The names of the repository/repositories and accession number(s) can be found below: https://www.ncbi.nlm.nih.gov/geo/, GSE39582, GSE29621, and GSE17536.

## Author Contributions

YR and WY conceived and designed the study. YR and SF performed the experiments and analyzed the data. YR and SH wrote the manuscript. WY contributed to manuscript revision. All authors contributed to the article and approved the submitted version.

## Funding

This study was supported by the Guangdong Natural Science Funds for Distinguished Young Scholar (No. 2017A030306030 to WY).

## Conflict of Interest

The authors declare that the research was conducted in the absence of any commercial or financial relationships that could be construed as a potential conflict of interest.

## Publisher's Note

All claims expressed in this article are solely those of the authors and do not necessarily represent those of their affiliated organizations, or those of the publisher, the editors and the reviewers. Any product that may be evaluated in this article, or claim that may be made by its manufacturer, is not guaranteed or endorsed by the publisher.

## Supplementary Material

Supplementary Figure S1 Kaplan-Meier survival subgroup analysis according to the signature stratified by clinical characteristics. **(A)** Age ≤ 70 years and age > 70 years. **(B)** Female and male. **(C)** T1-2 and T3-4. **(D)** N0 and N1-2. **(E)** M0 and M1. **(F)** Stage I–II and stage III–IV.

Supplementary Figure S2 Correlation between risk score and GSEA, somatic variation. **(A)** Enriched gene sets annotated by the KEGG collection between the high- and low-AAMRGs-risk groups in the cohort. **(B)** TMB levels between the high- and low-risk groups. **(C)** Correlation analysis between risk score and mutation load. **(D,E)** The mutation rates of reported prognostic-related genes in low- and high-risk groups.

Supplementary Figure S3 Differential chemotherapeutic response based on IC50 available between the high- and low-risk groups. **(A–F)** The half-maximal inhibitory concentration (IC50) of six chemotherapeutic agents (Paclitaxel, Metformin, Rapamycin, Bortezomib, Sorafenib, Gemcitabine).

## Abbreviations

COAD: colon adenocarcinoma
GEO: Gene Expression Omnibus
TCGA: The Cancer Genome Atlas
AAMRGs: amino acid metabolism-related genes
MSI: microsatellite instability
TPM: transcripts per million
LASSO: least absolute shrinkage and selection operator
ROC: receiver operating characteristic
TCIA: The Cancer Immunome Atlas database
OS: overall survival
AUC: area under the curve
HR: Hazard ratio
TMB: tumor mutation burden
GSEA: Gene Set Enrichment Analysis.
