# Supplementary figures and images for: A Prognostic Model for Colon Adenocarcinoma Patients Based on Ten Amino Acid Metabolism Related Genes
Source: Front Public Health. 2022 May 27;10:916364. doi: 10.3389/fpubh.2022.916364 (PMC9197389; doi:10.3389/fpubh.2022.916364)

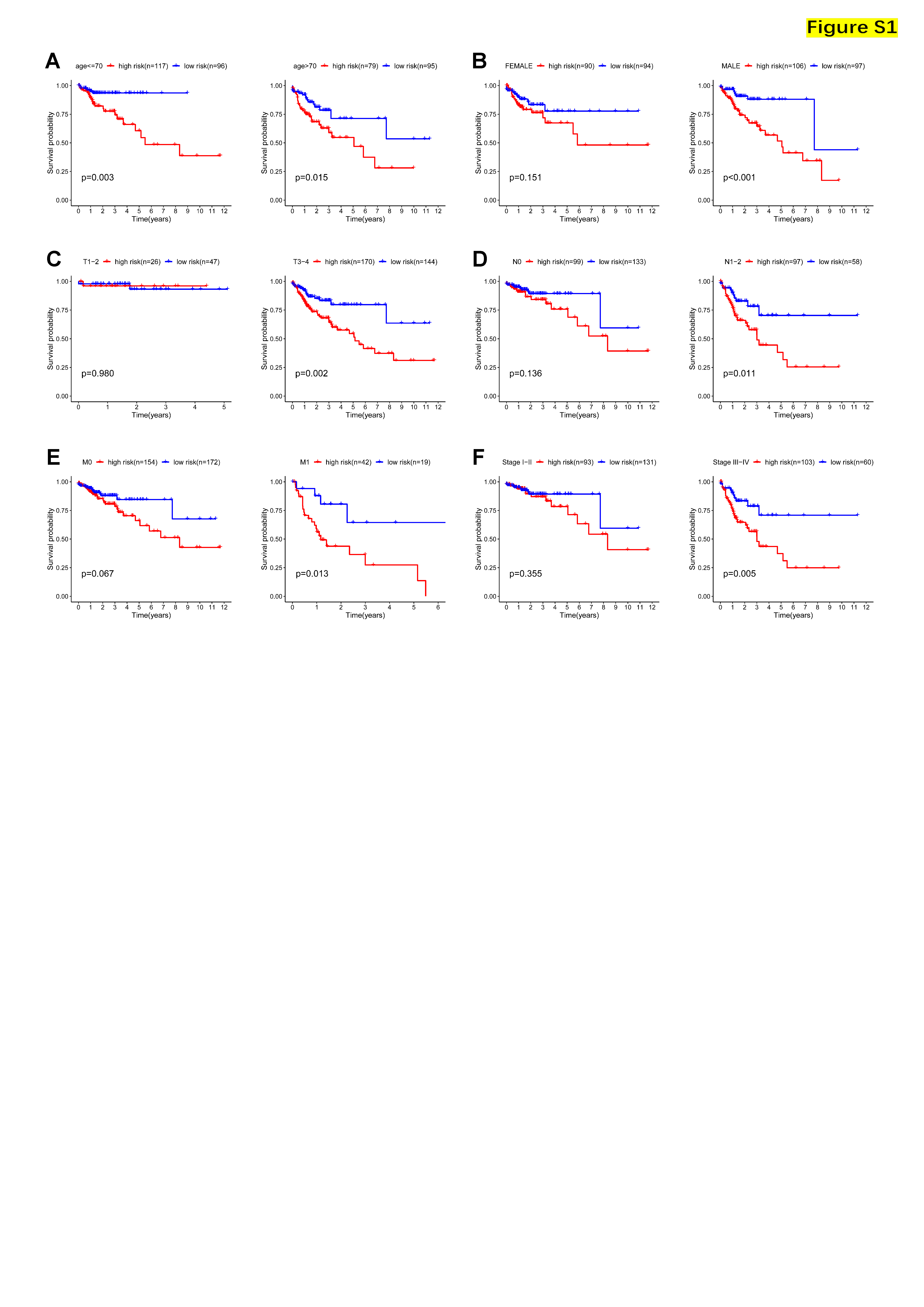

Supplement: Supplementary Figure S1 — Kaplan-Meier survival subgroup analysis according to the signature stratified by clinical characteristics. (A) Age ≤ 70 years and age > 70 years. (B) Female and male. (C) T1-2 and T3-4. (D) N0 and N1-2. (E) M0 and M1. (F) Stage I–II and stage III–IV. [file Image_1.TIFF]

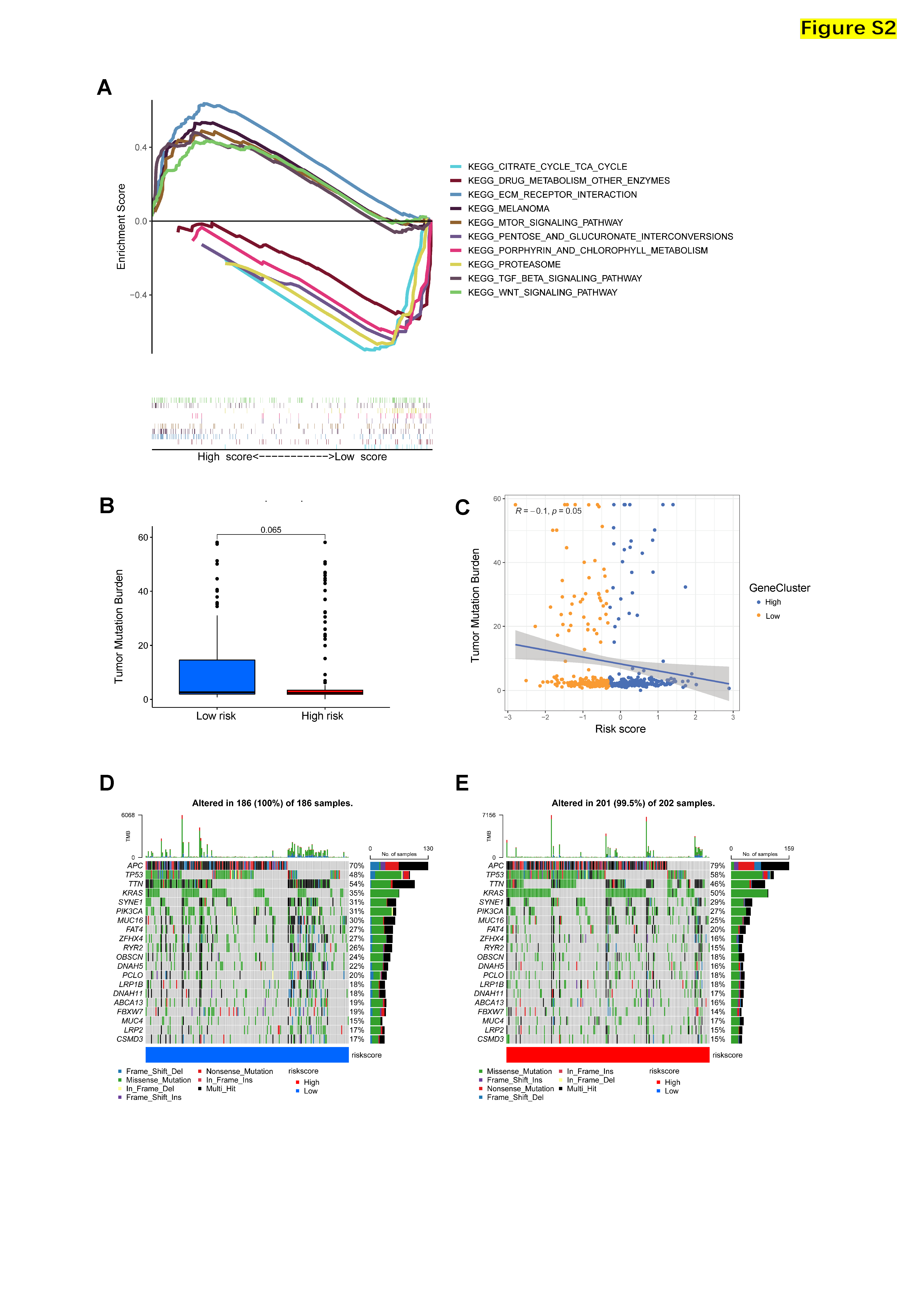

Supplement: Supplementary Figure S2 — Correlation between risk score and GSEA, somatic variation. (A) Enriched gene sets annotated by the KEGG collection between the high- and low-AAMRGs-risk groups in the cohort. (B) TMB levels between the high- and low-risk groups. (C) Correlation analysis between risk score and mutation load. (D,E) The mutation rates of reported prognostic-related genes in low- and high-risk groups. [file Image_2.TIFF]

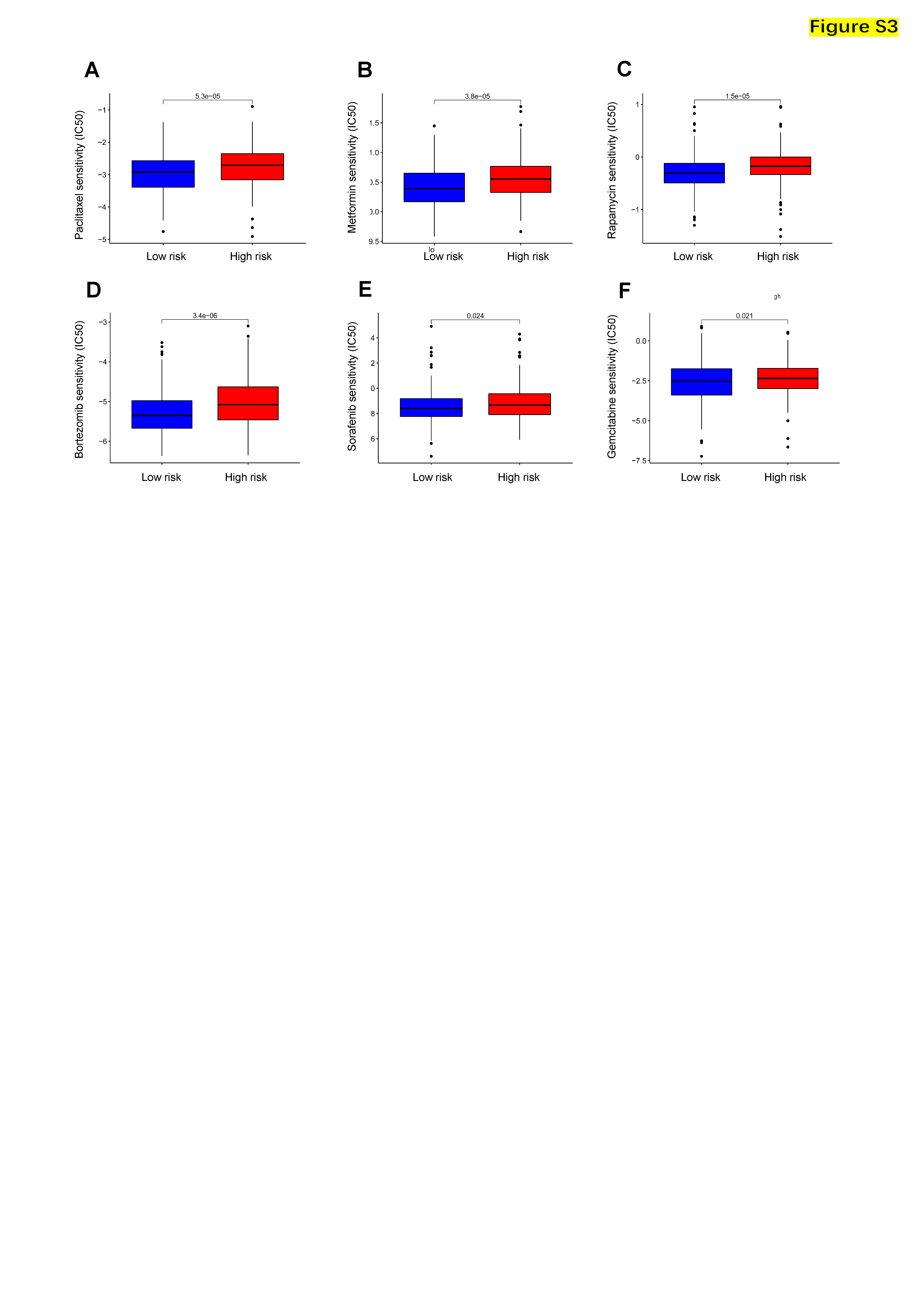

Supplement: Supplementary Figure S3 — Differential chemotherapeutic response based on IC50 available between the high- and low-risk groups. (A–F) The half-maximal inhibitory concentration (IC50) of six chemotherapeutic agents (Paclitaxel, Metformin, Rapamycin, Bortezomib, Sorafenib, Gemcitabine). [file Image_3.TIFF]
